# Mitochondrial Damage-Associated Molecular Patterns (MTDs) Are Released during Hepatic Ischemia Reperfusion and Induce Inflammatory Responses

**DOI:** 10.1371/journal.pone.0140105

**Published:** 2015-10-09

**Authors:** Qianni Hu, Caroline Ruth Wood, Sanem Cimen, Ananda Baskaran Venkatachalam, Ian Patrick Joseph Alwayn

**Affiliations:** 1 Department of Pathology, Dalhousie University, Halifax, Nova Scotia, Canada; 2 Department of Surgery, Dalhousie University, Halifax, Nova Scotia, Canada; 3 Departments of Surgery, Pathology, Microbiology & Immunology, Dalhousie University, Halifax, Nova Scotia, Canada; Universidade de Sao Paulo, BRAZIL

## Abstract

Ischemia / reperfusion injury (IRI) during the course of liver transplantation enhances the immunogenicity of allografts and thus impacts overall graft outcome. This sterile inflammatory insult is known to activate innate immunity and propagate organ damage through the recognition of damage-associate molecular pattern (DAMP) molecules. The purpose of the present study was to investigate the role of mitochondrial DAMPs (MTDs) in the pathogenesis of hepatic IRI. Using *in vitro* models we observed that levels of MTDs were significantly higher in both transplantation-associated and warm IR, and that co-culture of MTDs with human and rat hepatocytes significantly increased cell death. MTDs were also released in an *in vivo* rat model of hepatic IRI and associated with increased secretion of inflammatory cytokines (TNF-α, IL-6, and IL-10) and increased liver injury compared to the sham group. Our results suggest that hepatic IR results in a significant increase of MTDs both *in vitro* and *in vivo* suggesting that MTDs may serve as a novel marker in hepatic IRI. Co-culture of MTDs with hepatocytes showed a decrease in cell viability in a concentration dependent manner, which indicates that MTDs is a toxic mediator participating in the pathogenesis of liver IR injury.

## Introduction

Ischemia reperfusion injury (IRI) is a phenomenon whereby cellular damage in a hypoxic organ is exacerbated following the restoration of oxygen delivery [1]. On the cell-based IRI, this process can be divided into two steps: first, cells subjected to combined oxygen-nutrition deprivation; second followed by re-oxygenation. The liver, an organ with high energy requirements, is highly dependent on oxygen supply and susceptible to hypoxic or anoxic conditions [2]. During liver transplantation, the liver undergoes a period of cold ischemia when the organ is retrieved from the donor and is stored under hypothermic conditions (termed cold ischemia). Following this, the liver is exposed to warm ischemia, when it is removed from cold storage, but before it is reperfused in the recipient. IRI in the context of liver transplantation is thus a combination of cold and warm ischemia followed by reperfusion (transplantation-associated ischemia/reperfusion) [1]. Isolated warm IRI occurs during elective liver surgery, trauma and incidental events such as hypovolemic shock. The pathophysiology of liver IRI includes direct cellular damage, such as hepatocyte necrosis and apoptosis, as the result of the ischemic insult as well as delayed organ dysfunction from activation of the innate immune system and propagation of the inflammatory response [3–7]. The ongoing donor organ shortage has led to the increased use of extended criteria donor organs, i.e. those from older or steatotic donors, or from donors after cardiocirculatory death, as well as organs that have been subjected to prolonged periods of cold ischemia. Unfortunately, these organs are more susceptible to IRI. In liver transplantation, IRI is the major contributor to posttransplant liver graft dysfunction, patient morbidity, and mortality [8–11]. To date, no specific treatment is available to prevent or reduce hepatic IRI and current management is based on supportive care. Thus, an improved understanding of the mechanisms underlying hepatic IRI may lead to more effective therapeutic interventions to minimize liver dysfunction and improve transplant outcomes.

A key contributor to the induced inflammatory response in IRI is the activation of endogenous ‘danger signals’ known as damage-associated molecular patterns (DAMPs), which are released from damaged tissues or necrotic cells. DAMPs are capable of initiating an inflammatory response analogous to pathogen-associated molecular patterns (PAMPs) of infectious pathogens [12]. Recently, several studies have described an association between the release of DAMPs during ischemia and subsequent damage to cells, activation of inflammatory responses and disruption of the tissue matrix [13,14]. To date, several endogenous DAMPs are elevated and contribute to poor outcome during liver injury including the high mobility group box-1 (HMGB-1) [13,15], the cytoplasmic Ca^2+^ regulator S100 [16], the cell matrix component hyaluronic acid [17], urate [18], ATP [18] and DNA [19].

We have focused on mitochondrial DAMPs (MTDs). Mitochondria are a source of DAMPs, a characteristic that stems from their bacterial ancestry [20]. MTDs express at least two molecular signatures, formyl peptides and mitochondrial DNA (mtDNA) that act on formyl peptide receptors (FPRs) and Toll-like receptors (TLRs), respectively [21,22]. There are evolutionarily conserved similarities to bacterial PAMPs, and the release of MTDs has been shown to lead to a “sepsis-like” inflammation state [23]. Although previous studies have investigated the ability of circulating nuclear fragment DNA and HMGB-1 protein to mediate organ damage in hepatic IR injury through TLRs [24,25], the involvement of MTDs, which are closely associated with TLRs, in these processes has not yet been examined. Traditionally, mitochondria have a key role in ATP synthesis; in addition, they act as crucial transducers and effectors in multiple processes including cell death signaling, oxidant signaling, innate immunity, Ca^2+^ homeostasis, fatty acid synthesis and autophagy [16]. Recent reports have examined the role of released fragments from mitochondria in the genesis and maintenance of cardiovascular diseases [26]. Most importantly, Zhang *et al*. identified circulating MTDs as major mediators of the systemic inflammatory response syndrome (SIRS) in trauma patients [23]. Increasing evidence suggests that MTDs function as DAMPs in the plasma of acetaminophen overdose patients [27], patients in the intensive care unit [28], patients with pediatric traumatic brain injury [29] and that blood products containing MTDs might be a potential effector of transfusion-related acute lung injury [30].

In the present study, we sought to understand the role of MTDs in the pathogenesis of hepatic IRI. The first aim of this study was to determine whether MTDs are released during hepatic IRI. We used *in vitro* models of cold and warm IR and confirmed the obtained findings with an *in vivo* model of hepatic IR. The second aim was to investigate whether MTDs can induce hepatocyte damage, irrespective of IR. We demonstrated that both in transplantation and warm IR *in vitro* models MTDs levels were significantly elevated. Furthermore, following IR *in vivo*, MTDs were also significantly elevated. Finally, co-culture of MTDs with hepatocytes showed a decrease in cell viability in a concentration dependent manner. This study reveals a dominant role for MTDs in the pathogenesis of hepatic IRI and demonstrates its potential as a biomarker of injury following IR.

## Materials and Methods

### Animals

Male Lewis rats (250–300 g) were purchased from Charles River, Sherbrooke, Canada. Rats were housed 2 per cage in a pathogen-free facility with a 12-h light-dark cycle. Water and food were available ad libitum. This study was carried out in strict accordance with the recommendations in the Guide for the Care and Use of Laboratory Animals issued by the Canadian Council on Animal Care. The experimental protocol was approved by the Animal Care Committee of Dalhousie University (Protocol number: 12–088).

### Cell culture

The McA-RH7777 rat hepatocyte cell line and Hep G2 human hepatocyte cell line were purchased from American Type Culture Collection (ATCC) (Manassas, VA, USA) and cultured in Dulbecco's Modified Eagle's Medium (DMEM) medium supplemented with 10% heat-inactivated FBS, 2mM L-glutamin, 100U/ml penicillin and 100mg/ml streptomycin in a humidified 5% CO_2_ atmosphere.

### Ischemia/reperfusion (IR) *in vitro* models

For exposure to IR, culture dishes were placed in a Modular Incubator Chamber (MIC-101, Billups-Rothenberg. Inc., Del Mar, CA), functioning as an airtight chamber according to the manufacturer’s instructions. To simulate cold IR, hepatocytes were cultured in University of Wisconsin (UW) cold preservation solution at 4°C incubator for 6 h. Reperfusion was then simulated by changing the UW solution to warm DMEM completed medium, removing the plates from the hypoxic chamber and placing them in a normoxic, humidified incubator for another 1 h. To mimic warm IR alone *in vitro*, cells were cultured in the DMEM in the hypoxic chamber at 37°C for 2 h, then reoxygenation achieved by the same way as transplantation IR *in vitro*. Rat hepatocytes (McA RH-7777) and human hepatocytes (HepG2) were investigated in the *in vitro* models described above, respectively: (1) Control group (2) Ischemia group (3) Ischemia reperfusion (IR) group.

### Total hepatic warm IR

Total hepatic IR was performed as previously described with minor modifications [31]. In brief, rats underwent a laparotomy and a sterile atraumatic clip was placed on the portal triad for 60 min to induce total hepatic ischemia and mesenteric congestion. Reperfusion was initiated by removal of the clamp. Animals were randomly allocated to two groups (n = 3 each): (1) Control group undergoing a sham operation, laparotomy without clamping of the portal triad and (2) Experimental group undergoing *in vivo* IR as described. 100μl blood was drawn from the right atrium of the heart catheter every 30 min through the inferior vena cava (IVC). The rectal temperature was maintained at 37°C throughout surgery by a warming pad. Sham controls underwent anesthesia, laparotomy, and exposure of the portal triad without vascular occlusion. At the end of the predetermined period following reperfusion, the animals were euthanized by cervical dislocation for tissue and plasma collection.

### Real-time PCR

The primers for the real-time PCR analysis of mtDNA were: Primers for human cytochrome B (forward 5’-ATGACCCCAATACGCAAAAT-3’ and reverse 5’-CGAAGTTTCATCATGCGGAG-3’), human cytochrome C oxidase subunit III (forward 5’-ATGACCCACCAATCACATGC-3’ and reverse5’-ATCACATGGCTAGGCCGGAG-3’), human NADH dehydrogenase (forward 5’-ATACCCATGGCCAACCTCCT-3’ and reverse 5’-GGGCCTTTGCGTAGTTGTAT-3’), human β-actin (forward 5’-AGCGGGAAATCGTGCGTG-3’ and reverse 5’- CAGGGTACATGGTGGTGCC-3’), rat cytochrome B (forward 5’-TCCACTTCATCCTCCCATTC-3’ and reverse 5’-CTGCGTCGGAGTTTAA TCCT-39), rat cytochrome C oxidase subunit III (forward 5’-ACATACCA AGGCCACCAAC-3’ and reverse 5’-CAGAAAAATCCGGCAAAGAA-3’), rat NADH dehydrogenase (forward 5’-CAATACCCCACCCCCTTATC-3’ and reverse 5’-GAGGCTCATCCCGATCATAG-3’) and rat GAPDH (forward 5’-GAAATCCCCTGGAGCTCTGT-3’ and reverse 5’-CTGGCACCAGATGAAATGTG-3’) were synthesized by Integrated DNA Technologies (IDT, Coralville, US). The following primers were used to determine mRNA expression levels: TLR2, 5’-GGGATACAGGCCGTCAAGAC-3’ (forward) and 5’-CAGGAGCAGATGAAATGGTTGT-3’ (reverse); TLR4, 5’-CGCTCTGGCATCATCTTCAT-3’ (forward) and 5’-CTCCTCAGGTCAAAGTTGTTGC-3’ (reverse); TLR9, 5’-CCTGGCACACAATGACATTCA-3’ (forward) and 5’-TAAAGGTCCTCCTCGTCCCA-3’ (reverse); MyD88, 5’-GAGATCCGCGAGTTTGAGAC-3’ (forward) and 5’-TTGTCTGTGGGACACTGCTC-3’ (reverse); NF-κΒ, 5’-GAGGACTTGCTGAGGTTGG-3’ (forward) and 5’-TGGGGTGGTTGATAAGGAGTG-3’ (reverse); and glyceraldehyde phosphate dehydrogenase (GAPDH), 5’-GGCACAGTCAAGGCTGAGAATG-3’ (forward) and 5’-ATGGTGGTGAAGACGCCAGTA-3’ (reverse). Primer sequences have no significant homology with DNA found in any bacterial species published on BLAST. Samples that produced no PCR products after 40 cycles were considered ‘undetectable’ and the Ct number set to 40 for statistical purpose. All reactions were performed in a total volume of 20 μl containing 50 ng DNA and 1x SYBR Green. All reactions were done in duplicates and relative gene expression values determined using the 2^.ddCt^ method with Bio-rad iCycler iQ™ 5.

### Pathological evaluation

Liver samples were fixed with formalin and embedded with paraffin. Sections (5μm) were stained with haematoxylin-eosin (H&E). Pathological findings were assessed blinded to the group allocations. For each method used for detection of cellular damages, the number of injured cells was calculated using high power microscopy in 50 ± 5 fields (original magnification 10x) by morphological criteria (cell shrinkage, chromatin condensation, margination, apoptotic bodies, cell swelling, cell rupture).

### Immunohistochemistry for caspase-3

Apoptotic hepatocytes were identified using cleaved-caspase-3 / pro-caspase-3 immunohistochemistry. For caspase-3 immunostaining, 5μm-sections were dewaxed and hydrated through graded ethanols, cooked in 10 mM citrate buffer at pH 6.0 in a pressure cooker/antigen retriever at 125°C for 30 min (2100-Retriever, Electron Microscopy Sciences, Hatfield, PA, USA), then transferred into water to cool for 10 min. After 5 min of treatment in 3% H_2_O_2_, the slides were blocked with 10% normal goat serum (NGS) for 1 h. After blocking with 10% NGS, the slides were dried, and covered with the primary antibody (caspase-3 rabbit monoclonal antibody 1: 800 and pro-caspase-3 rabbit polyclonal antibodies 1:2000, Cell Signaling) overnight at 4°C. The slides were then washed three times and incubated with the secondary antibody (biotinylated goat anti-rabbit) for 1 hr. Following this, the slides were washed and dried, and then covered with ABC solution (Vector Laboratories, Burlingame, CA, USA) at room temperature for 1 h followed by the addition of DAB solution (Vector Laboratories) for 2 min. Later, DAB solution was washed and counterstained with Mayer’s Haematoxylin for 2 min followed by rinsing with tap water 2–3 times. The slides were then immersed in Scott’s solution for 2 min, and dehydrated and mounted with a coverslip.

### Preparation of MTDs

The Mitochondria Isolation Kit for Cultured Cells (Pierce) was used to isolate mitochondria from both rat and human hepatocytes, McA RH-7777 and Hep G2 according to the manufacturer's instructions. Isolated mitochondrial pellets were suspended in 1 ml of HBSS. Protease inhibitor cocktail (1:100) was added to the suspension. Because previous studies found significant amounts of circulating MTDs in trauma patients [23,29], and surmised that mechanical tissue injury and/or tissue necrosis was disrupting mitochondria to some extent *in vivo*, we standardized the experimental preparations with routine sonication on ice as previous described [23] at 100% amplitude (ten times, 30 s each time with 30 s intervals). The disrupted mitochondrial suspensions were then centrifuged at 12,000g for 10 min at 4°C followed by 100,000g at 4°C for 30 min. Residual supernatants were used for experiments. Protein concentrations of the MTD solutions were determined by BCA Protein Assay (Pierce).

### Apoptosis assay

Rat and human hepatocytes, McA RH-7777 and Hep G2, were labeled with fluorescein isothiocyanate (FITC) labeled Annexin V and propidium iodide (PI) using an Annexin V—FITC apoptosis detection kit (eBioscience) according to the manufacturer's instructions. Briefly, after 24h exposure to different concentrations of MTDs (0, 40, 100, 200, and 400 ug/ml), or after different treatments (Ischemia, IR, co-culture with mtDNA or MTDs) the cells were washed with cold PBS and then resuspended in 1X binding buffer. Aliquots of 2x10^5^ cells were mixed with 5 μl Annexin V—FITC and 10 μl PI for 10 min at room temperature in the dark. Fluorescence was detected within 1 h using flow cytometry.

### Liver enzyme levels

Serum (0.5 ml) was collected at 1 h post reperfusion and kept on ice until processed. Levels of aspartate transaminase (AST) and alanine transaminase (ALT) were determined in serum, in duplicate, using commercial kits (Sigma), according to the manufacturer’s protocols.

### Enzyme-Linked Immunosorbent Assay

Serum tumor necrosis factor alpha (TNF-α), interleukin (IL)-6, and IL-10 levels in rats were detected by an enzyme-linked immunosorbent assay (ELISA) kit (R&D System).

### Statistical analysis

Results are expressed as mean ± standard deviation. Differences between groups were assessed using the analysis of variance (ANOVA) with repeated measurements using the multiple comparison option of Duncan.

## Results

### Ischemia/reperfusion (IR) *in vitro* elevates mtDNA in both rat and human hepatocyte cell lines

Several nuclear DAMPs have been identified, including HMGB1 and nuclear fragment DNA, which are both elevated and contribute to poor outcomes in hepatic ischemia reperfusion [32,33]. The liver is one of the richest organs in terms of number and density of mitochondria. Many liver diseases are associated with the accumulation of damaged mitochondria [34]. To determine whether mitochondrial DAMPs play a role in hepatic organ damage after cold IR or warm IR, we used a Modular Incubator Chamber to simulate hypoxia at 4°C or at 37°C followed by transferring to a normoxic, humidified incubator at 37°C.

**Fig 1:**
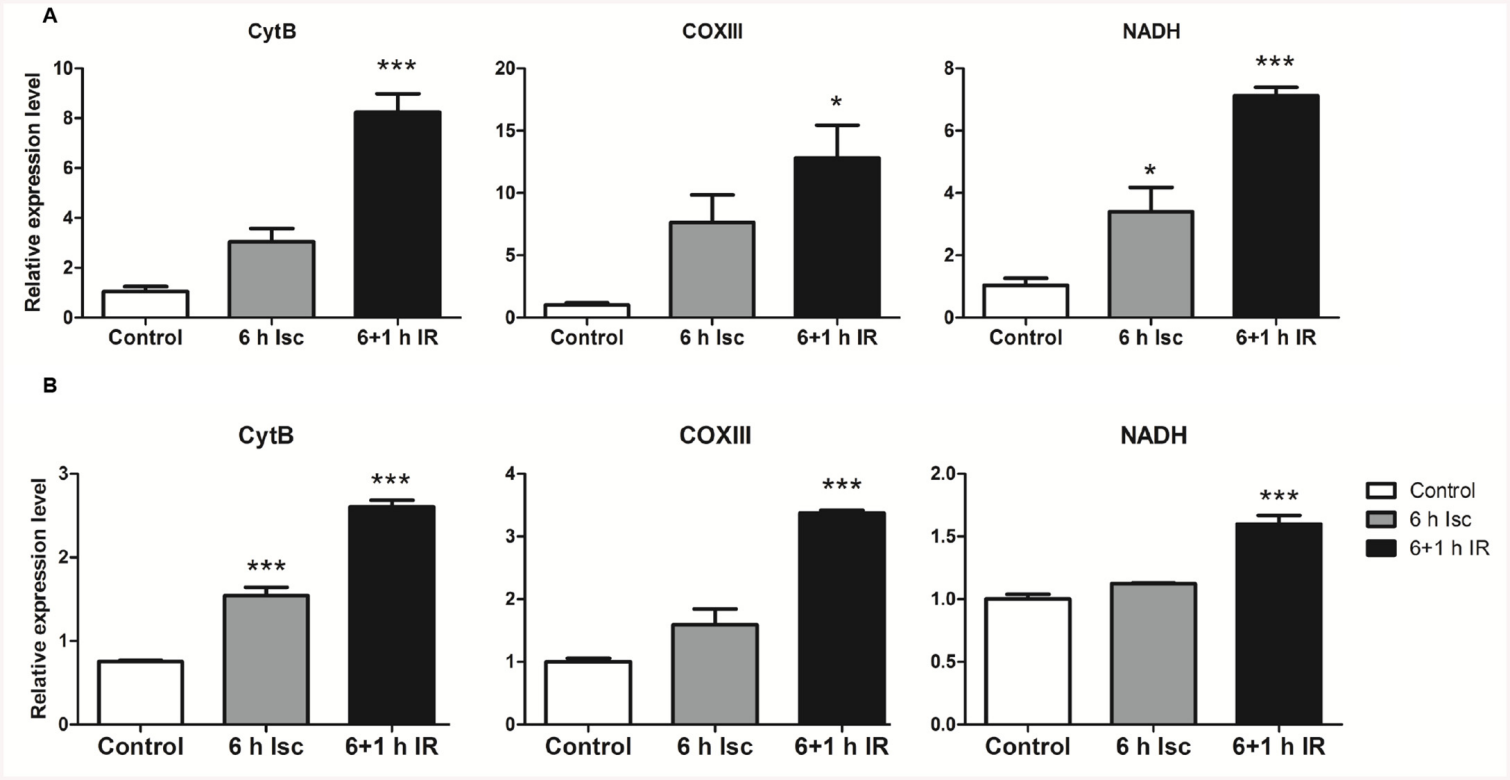
Transplantation-associated ischemia and warm reperfusion *in vitro* elevates mtDNA levels in both rat and human hepatocyte cell lines. (A) Six hours of transplantation-associated ischemia and one hour of reperfusion significantly increased the mtDNA levels in the rat hepatocyte cell line, McA RH7777. (B) Six hours transplantation-associated ischemia and one hour reperfusion significantly increased the mtDNA levels in the human hepatocyte cell line, HepG2. Control: cells were cultured in a conventional cell culture incubator for seven hours; 6 h Isc: six hours incubation in cold and ischemic conditions; 6+1 h IR: six hours incubation in cold and ischemic conditions followed by one hour of warm reperfusion; CytB, cytochrome B; COXIII, cytochrome C oxidase subunit III; NADH, NADH dehydrogenase. *, P = 0.01, **, P = 0.001, ***, P = 0.0001.

**Fig 2:**
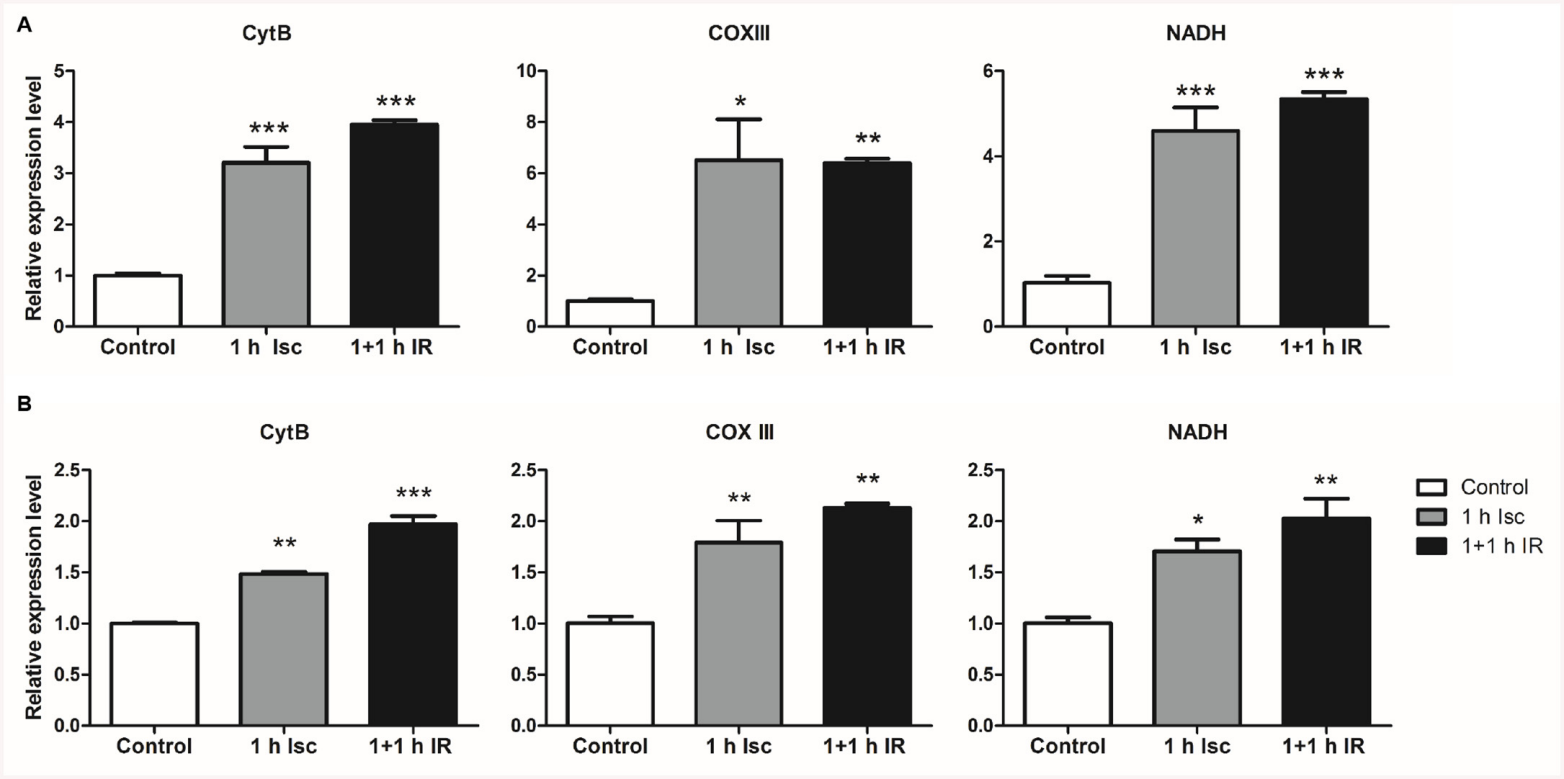
Warm ischemia and warm reperfusion *in vitro* elevates mtDNA levels in both rat and human hepatocyte cell lines. (A) One hour of warm ischemia and two hours of warm IR significantly increased the mtDNA levels in the rat hepatocyte cell line, McA RH7777. (B) One hour warm ischemia and two hours warm IR significantly increased the mtDNA levels in the human hepatocyte cell line, HepG2. Control, cells were cultured in normal cell culture incubator for two hours; 1h Isc, one hour warm ischemia incubation; 1+1 IR, one hour warm ischemia incubation followed by one hour warm reperfusion treatment, CytB, cytochrome B; COXIII, cytochrome C oxidase subunit III; NADH, NADH dehydrogenase. *, P = 0.01, **, P = 0.001, ***, P = 0.0001.

First, rat and human hepatocytes underwent cold ischemia for six hours in UW solution at 4°C, after which they were transferred to a normoxic, humidified incubator for another 1 h to simulate transplantation-related IR. We performed three independent experiments for both cell lines (rat hepatocyte, McA RH7777; human hepatocyte, HepG2). Cells that experienced cold IR *in vitro* showed significantly higher MTDs levels when compared to normoxic controls (Fig 1A and 1B). In a second set of experiments, cell lines were exposed to warm ischemia alone for one hour and then reperfusion for another hour. Both rat and human hepatocytes showed similar trends: there were significantly increased levels of MTDs following ischemia, which was maintained after reperfusion (Fig 2A and 2B). These results indicated that both cold and warm IR *in vitro* was associated with an elevation of MTDs.

### MTDs induce cell death *in vitro*

**Fig 3:**
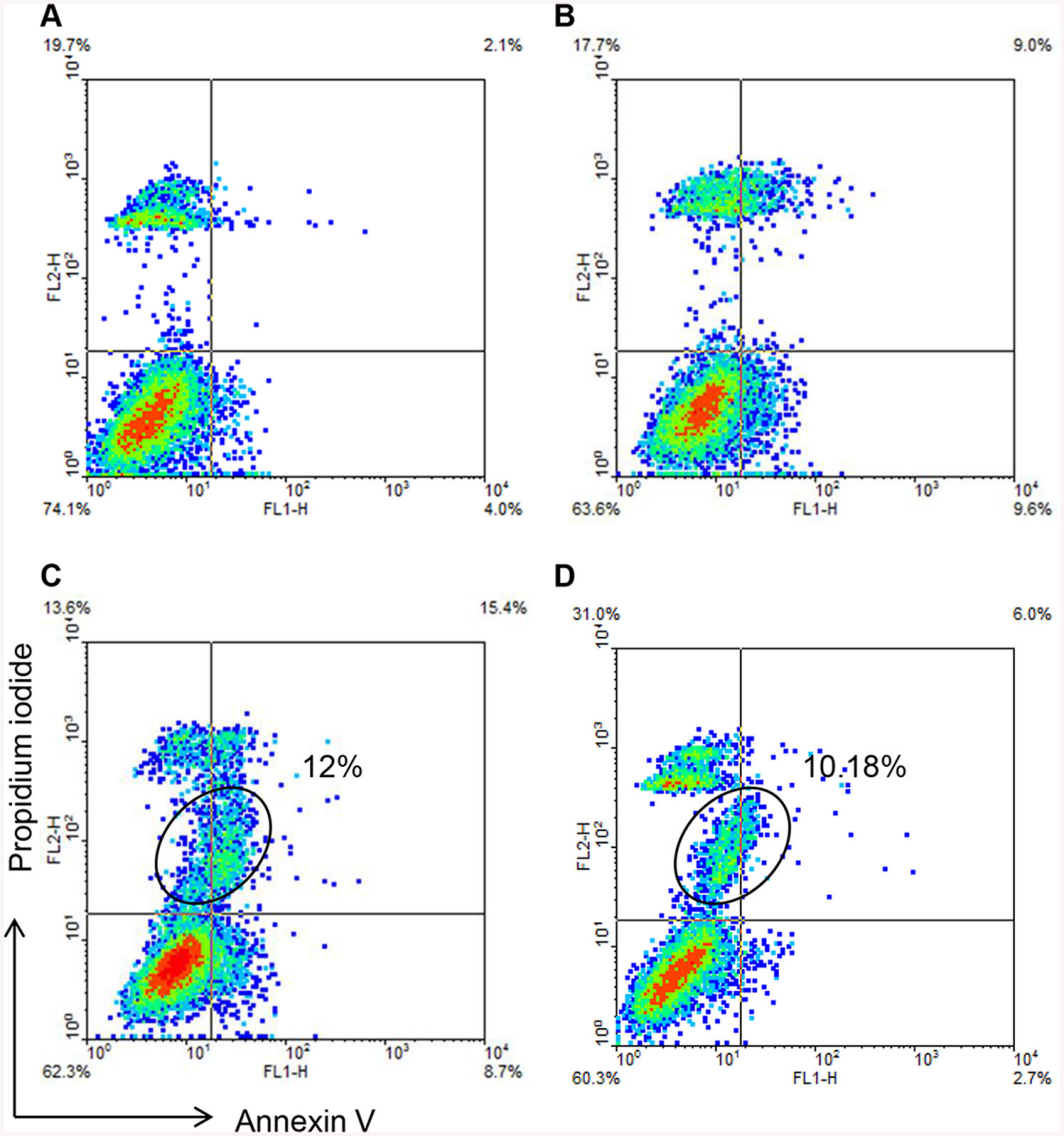
MTDs induce a similar pattern of cell death pattern compared to cells undergoing transplantation-associated ischemia and warm reperfusion *in vitro*. Apoptosis assay *in vitro* in HepG2 hepatocytes undergoing IR treatment and MTD incubation. (A) Control: HepG2 hepatocytes were cultured in normal environment. (B) Ischemia group: HepG2 hepatocytes that were maintained in a hypoxic environment for six hours *in vitro* demonstrated increased apoptosis when compared to Control cells. (C) IR group: HepG2 hepatocytes that were incubated in a hypoxic environment for six hours and subsequently transferred to a normoxic incubator for 24 hours demonstrated an increased percentage of both apoptosis and necrosis. In addition, these conditions induced a sub-population of PI-positive cells (gated in circle). (D) MTDs incubation group: HepG2 hepatocytes that were co-cultured with 400ug/ml MTDs in a normal environment for 24 hours demonstrated an increased percentage of apoptosis and necrosis with an increased sub-population of PI-positive cells (gated in circle), similar to the IR group (C). The figures shown are representative of three experiments with similar results. The PI single positive population is the basal level cell death.

**Fig 4:**
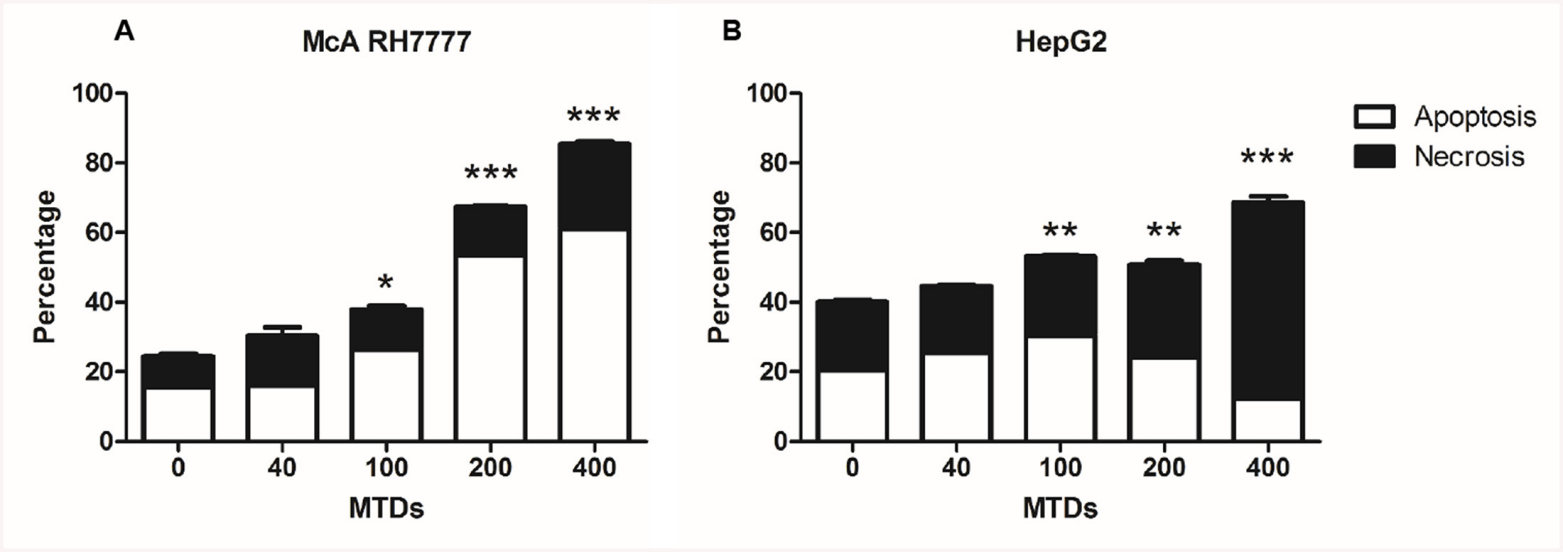
MTDs cause cell death *in vitro* in a dose dependent manner. (A) MTDs (Mitochondrial Damage-Associated Molecular Patterns) induce significant rat hepatocytes cell death at concentrations above 100 ug/ml, McA RH7777 co-cultured with different concentrations of MTDs (ug/mL) for 24 hours. (B) MTDs induce significant human hepatocytes cell death at concentrations above 100 ug/ml, HepG2 co-cultured with different concentrations of MTDs for 24 hours (ug/ml). The blank bar indicates Annexin V positive cells, the black bar indicates PI positive cells. *, P = 0.01, **, P = 0.001, ***, P = 0.0001 (n = 3/group).

To further explore the role of MTDs in IR, MTDs were extracted from hepatocyte cell lines, and co-cultured with each cell line in a normal, normoxic and normothermic environment. The Annexin V—FITC apoptosis detection kit (eBioscience) was used to detect apoptosis or necrotic cell death following exposure of normal hepatocytes to MTDs and cells undergoing IR treatments with controls. We found that ischemia alone and IR treatments *in vitro* can trigger cell death to different extents (Fig 3A–3C). MTDs can induce a similar pattern of cell death as IR treatment *in vitro*, as there was a subpopulation of PI positive cells in both of the treatments (Fig 3C and 3D). These results suggest that MTDs might exacerbate organ injury after IR. We subsequently incubated hepatocytes with different concentrations of MTDs, and observed that the MTDs trigger cell death in a dose dependent manner in both cell lines (Fig 4).

### Circulating MTDs increases in rat hepatic IR *in vivo*

**Fig 5:**
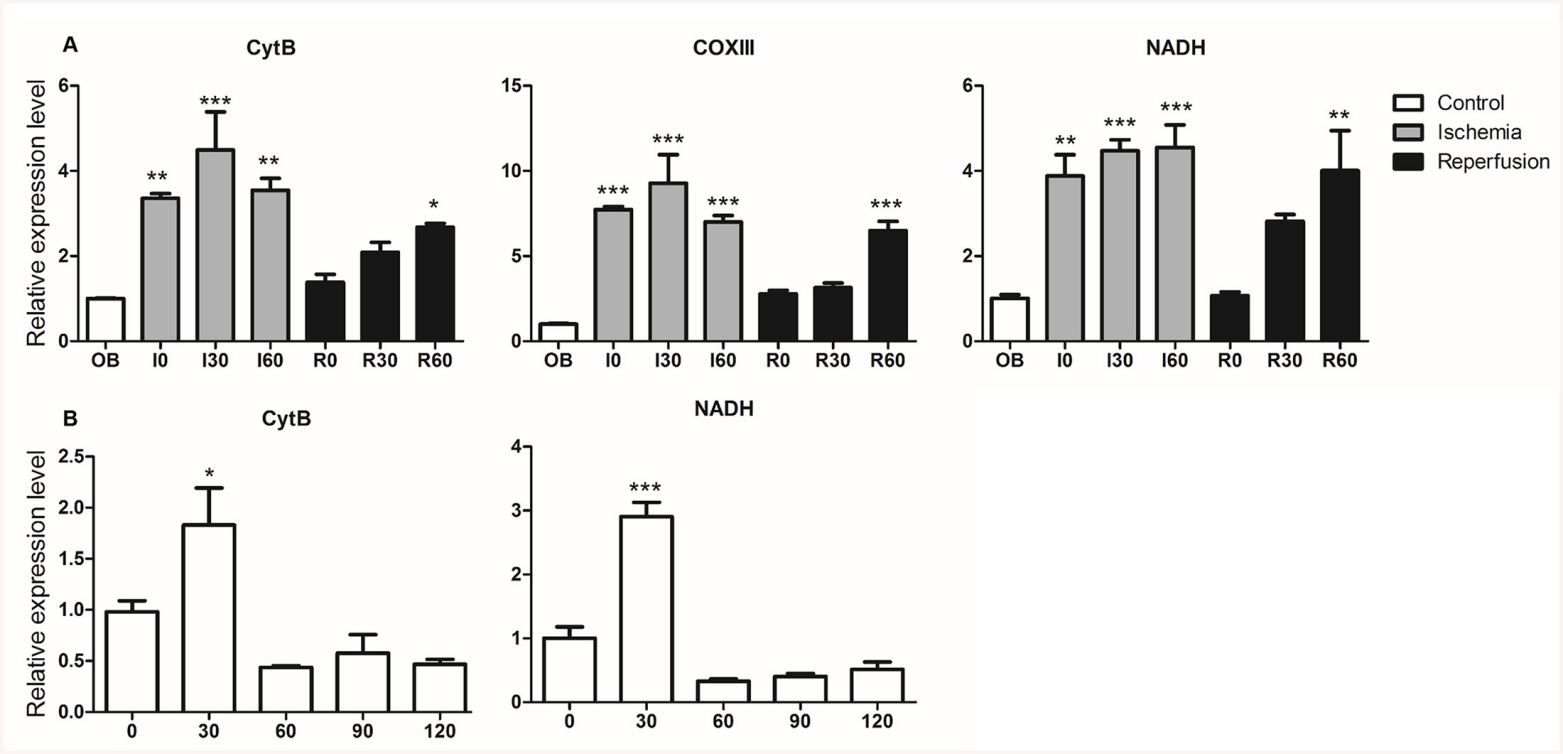
Hepatic IR increases mtDNA level in circulation *in vivo*. (A) One hour of warm ischemia and one hour of warm reperfusion *in vivo* significantly increased the mtDNA levels during both ischemia and IR; OB, blood collection immediately following laparotomy; I, ischemia: the atraumatic clip was placed on the portal triad for 60 minutes; R, reperfusion; reperfusion was initiated by removal of the atraumatic clip and observed for another 60 minutes; I0, I30, I60: ischemia for 0/30/60 minutes; R0, R30, R60: after 60 minutes portal triad clamping and the clip was removed to achieve reperfusion for 0/30/60 minutes. (B) mtDNA levels were not altered after 2 hours following a sham operation. 0, 30, 60, 90,120 indicate minutes of sham operating time, laparotomy without clamping of the portal triad. *, P = 0.01, **, P = 0.001, ***, P = 0.0001 (n = 3/group).

To investigate whether these *in vitro* results could be replicated in *vivo*, we assessed levels of MTDs in the blood of rats subjected to liver IR and sham. In our *in vivo* model, the IR group displayed a significant increase of MTDs over time, compared to the control group (Fig 5A). The MTDs levels of the control group also increased at 30min, but returned to baseline after 60min and remained stable during the next 60min (Fig 5B). Our results indicated that total hepatic IR can elicit a significant increase in circulating MTDs in blood, which may serve as a marker for hepatic IRI.

### Hepatic IR triggers liver injury *in vivo*

**Fig 6:**
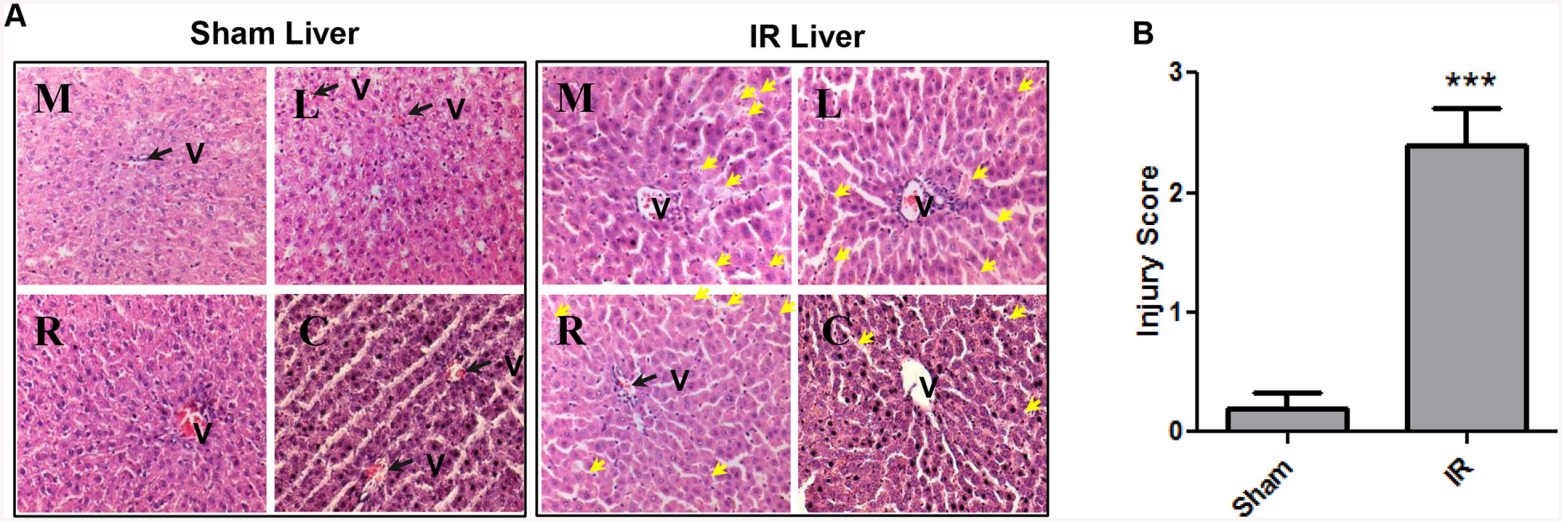
Histopathologic analyses of liver injury after IR. Liver sections were obtained from IR and sham control rats at the time point of 120 minutes. (A) IR induce hepatic injury assessed by H&E staining from different liver lobes: median (M), left (L), right (R) and caudate (C) lobes of sham and IR group, black arrow and V points to the portal vein, yellow arrows indicate necrotic cells. (B) IR induce significant hepatic injury assessed by Suzuki injury score. ***, P = 0.0001(n = 3/group).

**Fig 7:**
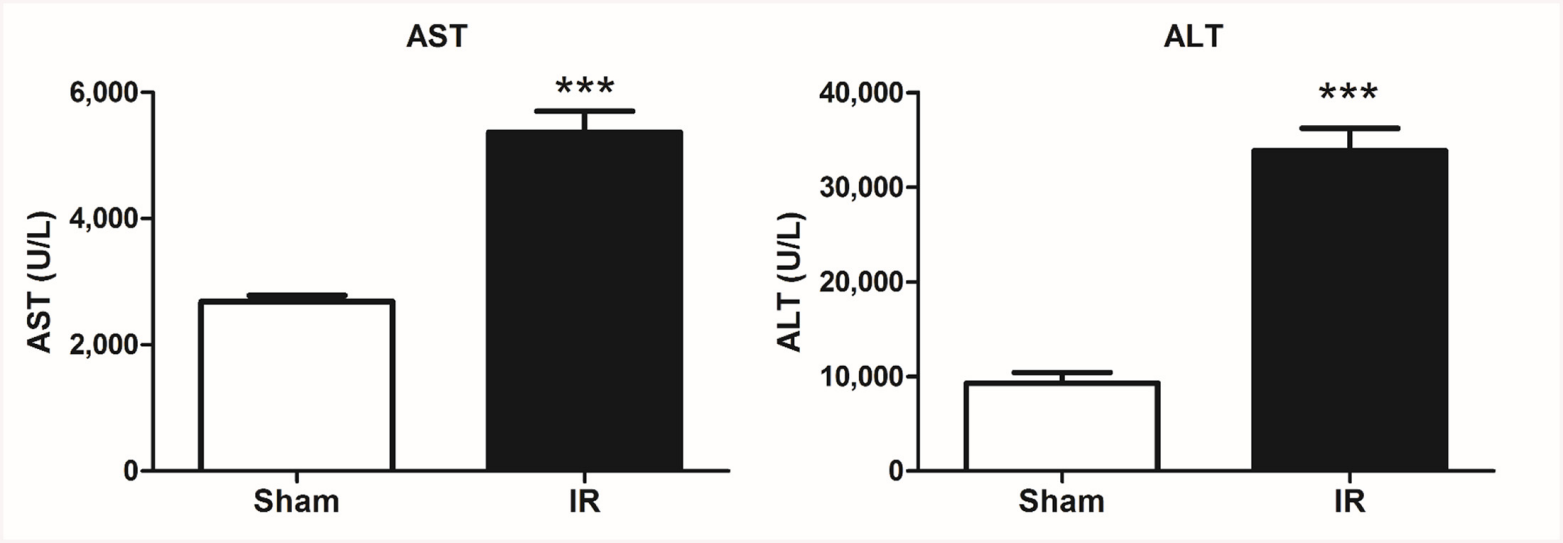
Hepatocellular injury evaluated by AST and ALT. IR induce significant hepatic injury as demonstrated by elevated AST and ALT levels. Serum samples were obtained from IR and sham rats at 120 minutes post reperfusion. A statistically significant difference was noted in mean serum ALT and AST levels following the induction of IR, compared with sham operated rats *in vivo*. ***, P = 0.0001(n = 3/group).

**Fig 8:**
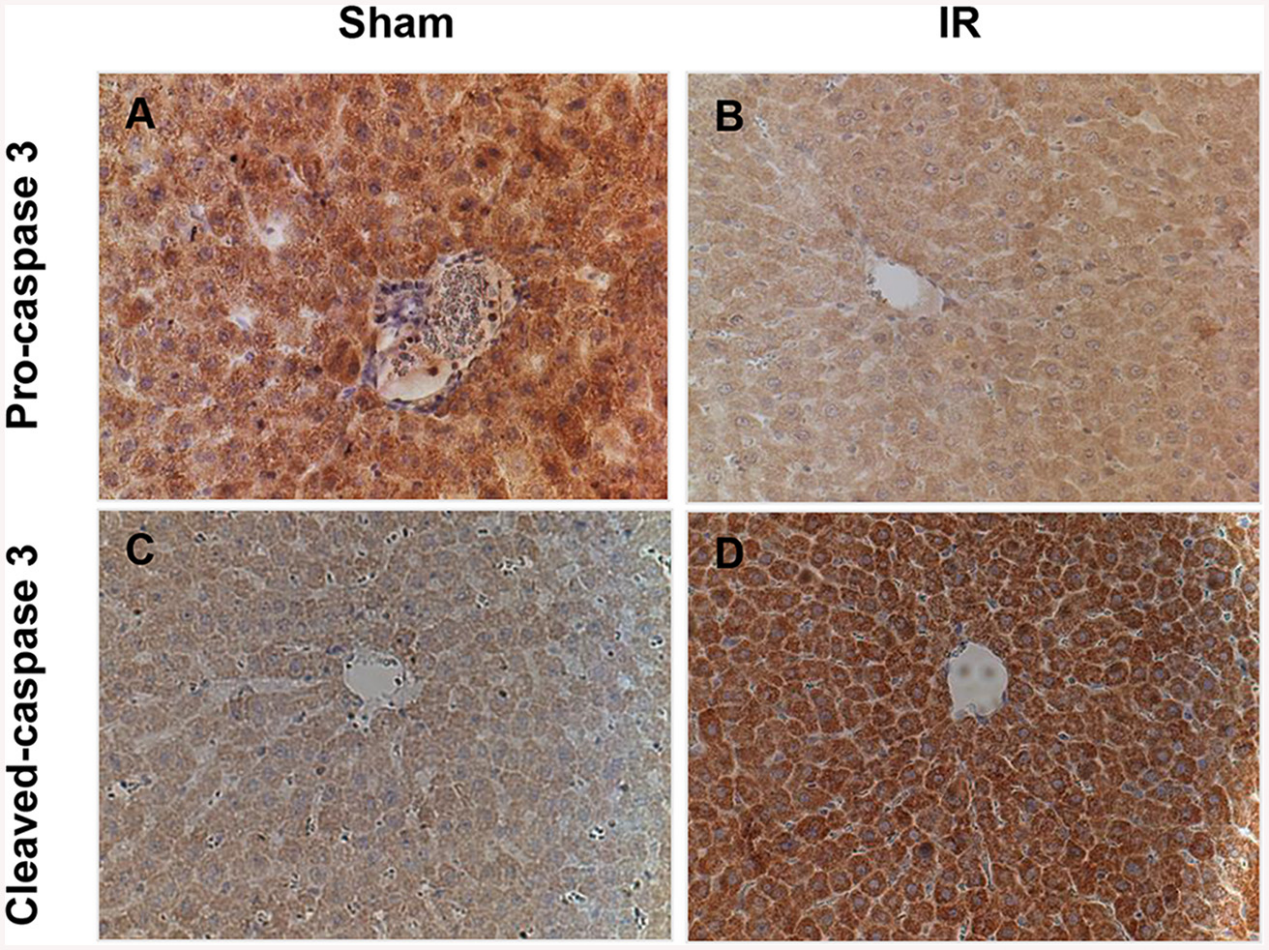
Total hepatic IR causes apoptosis *in vivo*. Liver sections were obtained from IR and sham control rats at 120 minutes post reperfusion. (A) and (B) the intensity of immunostaining of pro-caspase 3 significantly decreased after IRI compared with the sham group. (C) and (D) the intensity of immunostaining of cleaved-caspase 3 significantly increased after IRI compared with the sham group.

We next sought to confirm that total hepatic IR leads to injury of hepatocytes *in vivo*. We measured significantly more liver injury in the IR treatment group compared to Sham animals (Fig 6A and 6B). There were more necrotic cells with cell membrane disruption as indicated by the yellow arrows in Fig 6A. Liver enzyme levels remained within the normal range in the control, sham operated group for the duration of the study. A statistically significant difference was noted in mean serum ALT and AST levels following the induction of IR, compared with sham operated rats (Fig 7). Pro-caspase-3 is an intrinsic protein that is cleaved to segments when a cell undergoes apoptosis. Cleaved-caspase-3 is commonly used as an apoptotic marker [35]. We also assessed apoptosis in these groups with cleaved-caspase-3/ pro-caspase-3 immunohistochemistry (IHC). Liver sections from the IR group were highly positively for the cleaved form of caspase-3 compared to the sham group (Fig 8). In addition, we observed a trend that cleaved-caspase-3 accumulated while pro-caspase-3 reduced in the livers of the IR group, suggesting more apoptotic cells in these livers.

### Liver IR induced inflammatory cytokines production

**Fig 9:**
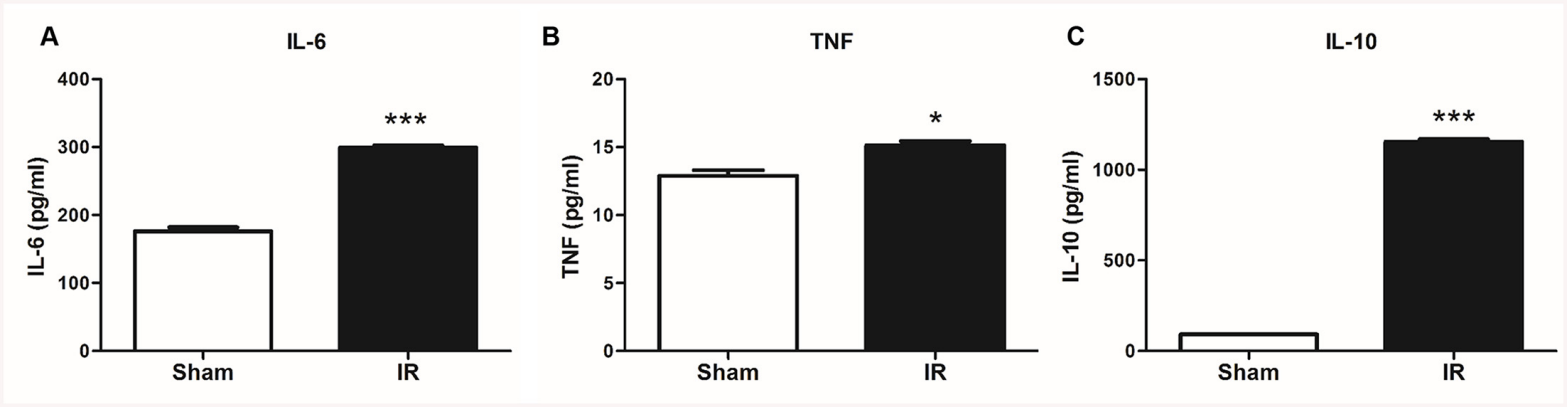
Total hepatic IR cause systemic inflammation in *in vivo*. Total hepatic IR induces significant IL-6 (A), TNF-α (B), and IL-10(C) cytokines release. Serum samples were obtained from IR and sham rats at 120 minutes post reperfusion. *, P = 0.01, ***, P = 0.0001 (n = 3/group).

We next determined whether these findings correlated with the production of inflammatory cytokines following IR. The serum levels of TNF-α, IL-6, and IL-10 were assessed by ELISA in the sham and IR groups. Among the cytokines examined, IL-6 and IL-10 production were found to be markedly increased in the serum after just one hour of hepatic IR (Fig 9A and 9C), whereas the TNF-α production showed slight increase when compared to the sham group (Fig 9B). The increases in IL-6, TNF-α, and IL-10 were associated with an increase in circulatory MTDs.

### MTDs induce *MyD88* and *NFκB* expression and up-regulate expression of *TLR2*, *TLR4* and *TLR9*

**Fig 10:**
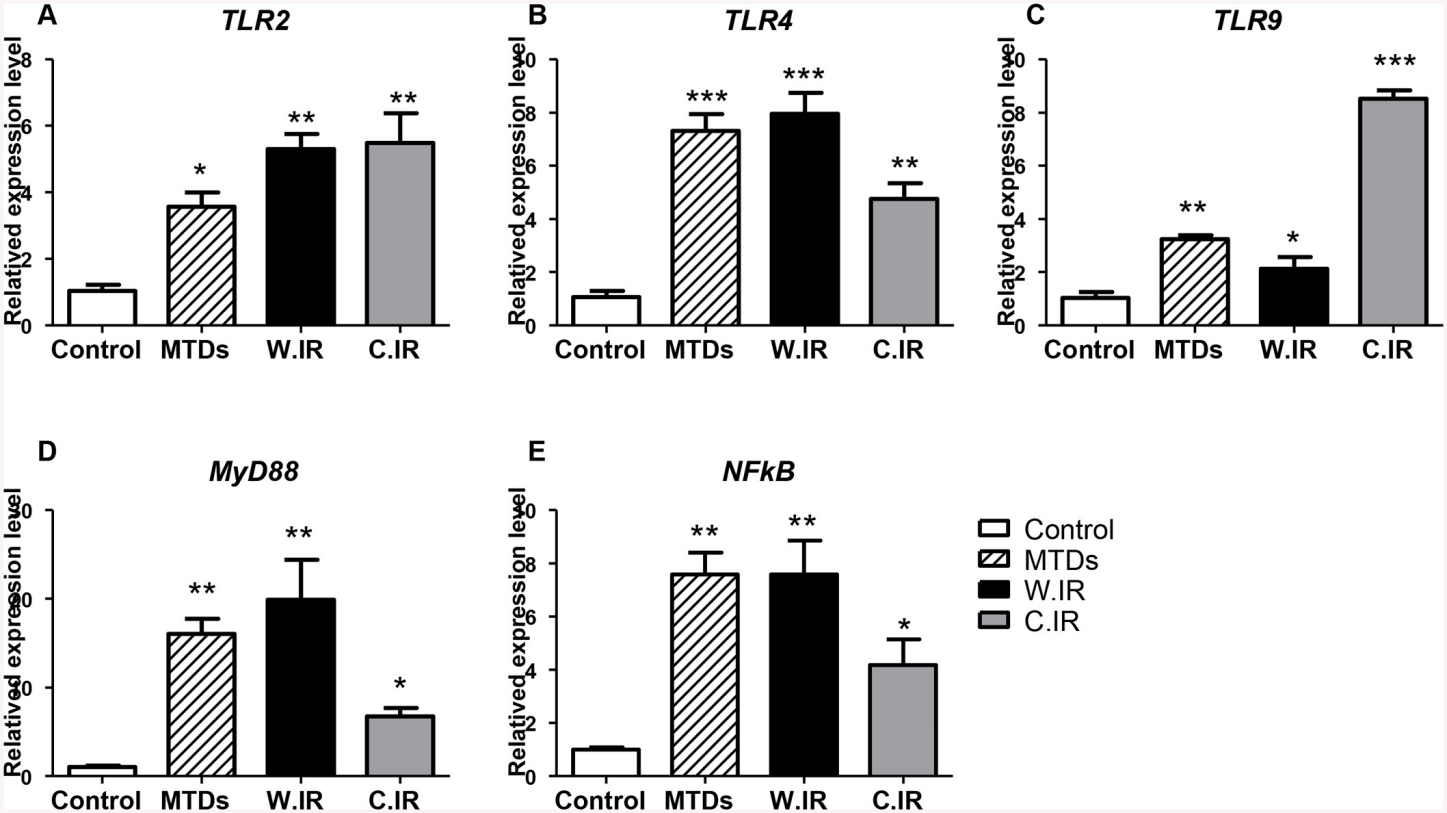
Fold changes in *TLR2*, *TLR4*, *TLR9*, *MyD88* and *NF-κB* mRNA levels. MTDs induce *MyD88* and *NFκB* expression and up-regulate expression of *TLR2*, *TLR4* and *TLR9* similar to warm hepatic IR. (A)-(E), The expression level of *TLR2*, *TLR4*, *TLR9*, *MyD88* and *NF-κB* significantly increased with MTDs co-culture, warm IR, and cold IR treatment compared to control; MTDs, co-culture MTDs with McA RH7777 hepatocyte cell line for 24 hours; W.IR, McA RH7777 hepatocyte cell line underwent warm ischemia for 1 hour followed by reperfusion for 24 hours *in vitro*; C.IR, McA RH7777 hepatocyte cell line underwent cold ischemia for 6 hours followed by reperfusion for 24 hours *in vitro*. *, P = 0.01, **, P = 0.001, ***, P = 0.0001 (n = 3/group).

Expression of mRNA for *TLR2*, *TLR4*, *TLR9*, *MyD88* and *NFκB* were similar for hepatocytes co-cultured with 400ug/ml MTDs, warm IR and cold IR treatments (Fig 10). The expression levels of all these genes were significantly higher in hepatocytes co-cultured with MTDs as well as the warm and cold IR treatments when compared to the control group. The expression levels, all normalized to the control group, are as follows, TLR2 (MTDs: 3.572±0.426, p<0.01; warm IR: 5.302±0.462, p<0.001; cold IR: 5.492±0.893, p<0.001), TLR4 (MTDs: 7.309±0.635, p<0.0001; warm IR: 7.953±0.794, p<0.0001; cold IR: 4.753±0.594, p<0.001), TLR9 (MTDs: 3.246±0.145, p<0.001; warm IR: 2.129±0.430, p<0.01; cold IR: 8.528±0.303, p<0.0001), MyD88 (MTDs: 16.04±1.718, p<0.001; warm IR: 19.90±4.494, p<0.001; cold IR: 6.744±0.977, p<0.01), and NFκB (MTDs: 7.591±0.802, p<0.001; warm IR: 7.589 ±1.266, p<0.001; cold IR: 4.176±0.970, p<0.01).

### MTDs induced hepatocytes up-regulate expression of TNFα

**Fig 11:**
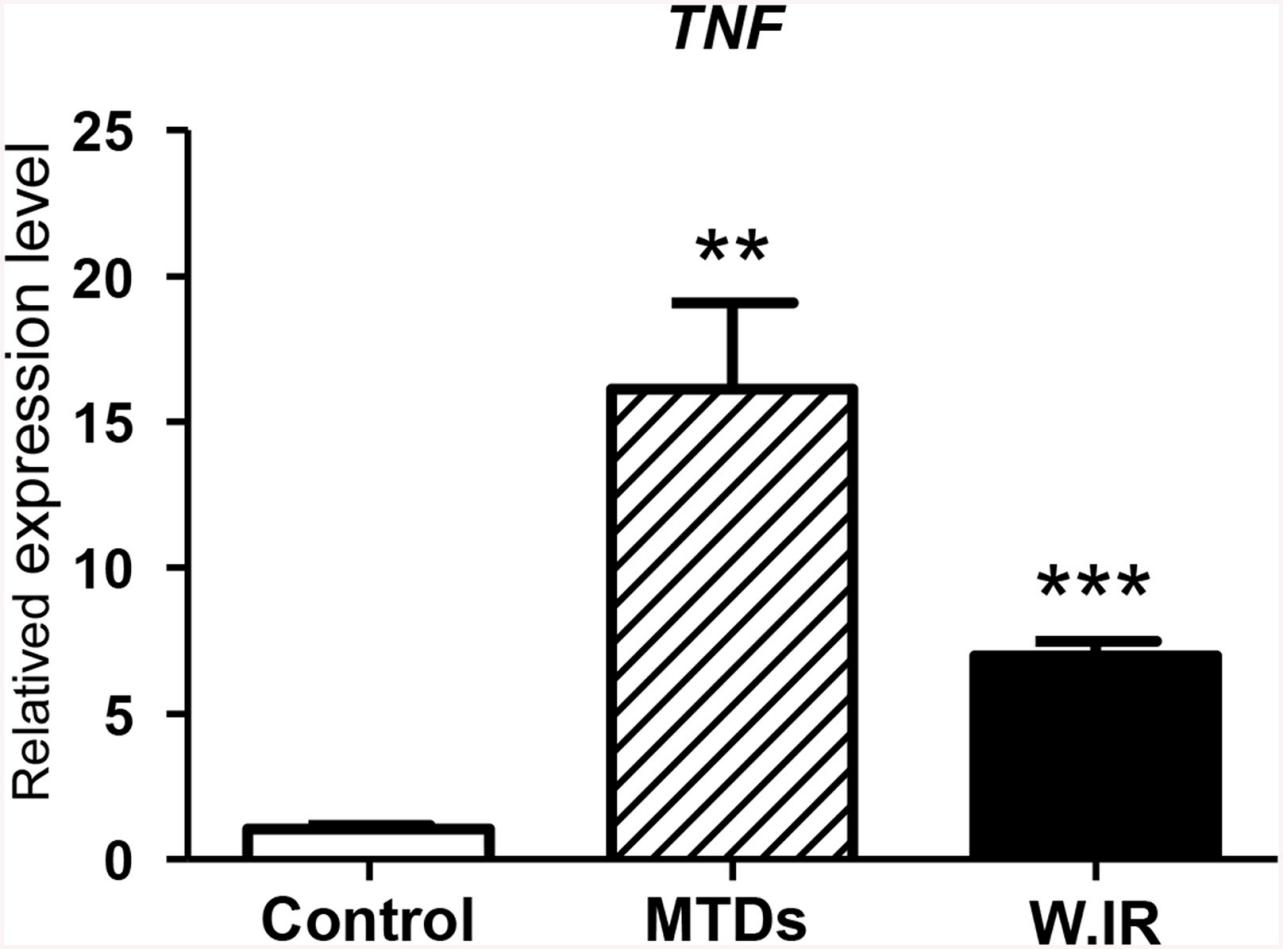
Fold changes in *TNFα* mRNA levels. MTDs-induced hepatocytes up-regulate expression of TNFα similar to warm IR treatment. MTDs, co-culture 400ug/ml MTDs with McA RH7777 hepatocyte cell line for 24 hours; W.IR, McA RH7777 hepatocyte cell line underwent warm ischemia for 1 hour and reperfusion for 24 hours *in vitro*. **, P = 0.001, ***, P = 0.0001 (n = 3/group).

We also assayed the pro-inflammatory cytokines, IL-6 and TNFα, in the supernatant by ELISA. IL-6 levels were not increased in the control, MTDs co-cultured or warm/cold IR groups (Data not shown). Interestingly, we found a slightly TNFα in the supernatant of both the MTDs co-cultured hepatocytes (20.23±6.971pg/ml) and the cells subjected to warm IR (6.799±3.519pg/ml), whereas these levels were undetectable in the control group and cells subjected to cold IR (Table 1). We confirmed that the TNFα expression level was significantly up-regulated in hepatocytes co-cultured with MTDs by qPCR (Fig 11), which was 16.12±2.974 fold higher than the control group (P<0.001).

**Table 1:** TNFα was released by the hepatocytes in MTDs co-cultured and warm IR.

	Control	MTDs*	Warm IR	Cold IR
TNFα	NA	20.23±6.971	6.799±3.519	NA

* MTDs, co-culture 400ug/ml MTDs with McA RH7777 hepatocyte cell line for 24 hours; W.IR, McA RH7777 hepatocyte cell line underwent warm ischemia for 1 hour followed by reperfusion for 24 hours *in vitro*; C.IR, McA RH7777 hepatocyte cell line underwent cold ischemia for 6 hours followed by reperfusion for 24 hours *in vitro*. NA, not available.

## Discussion

An emerging body of evidence indicates that endogenous DAMPs play critical role in the initiation of hepatic ischemia reperfusion injury. MTDs express at least two molecular signatures, formyl peptides and mitochondrial DNA (mtDNA) that act on formyl peptide receptors (FPRs) and Toll-like receptors (TLRs), respectively [21,22]. In this study, we demonstrated that MTDs were elevated in hepatocytes following IR *in vitro* and in the circulation after hepatic warm IR *in vivo* (Figs 1, 2 and 5). These results suggest that MTDs may serve as a marker for hepatic IR injury.

Interestingly we found that there were different patterns of MTD release when cells were subjected to transplantation-related and warm ischemia. The elevation of MTDs was markedly higher in the IR compared with cold ischemia incubation alone group in both hepatocyte cell lines (Fig 1). In contrast, after warm ischemia, the levels of MTDs increased immediately both *in vitro* and *in vivo*, and maintained these high levels in the reperfusion phases (Figs 2 and 5). These results indicate that different mechanisms of injury are likely involved in cold and warm hepatic IRI. This is consistent with previous studies demonstrating that in cold ischemia, during hypothermic preservation, toxic mediators are not released [36], whereas during warm ischemia, these toxic mediators are up-regulated [37]. In Fig 5, we noticed a decrease in MTDs at R0 (immediately after release of the clamp), and subsequent increase in MTDs over time during the next 60min of reperfusion. This was likely due to dilution of MTDs by the reperfused blood to the liver. The increase of MTDs after reperfusion suggests not only that warm ischemia induces MTDs elevation, but that reperfusion further triggers additional MTDs release.

We further showed that MTDs could trigger apoptosis and necrosis in a dose dependent manner *in vitro* (Fig 4). These results suggest that MTDs may serve as a toxic mediator participating in the pathogenesis of liver IR injury. Zhang *et al*. [23] reported that severe trauma releases mtDNA and formyl peptides into the circulation and that MTDs was further elevated 24h after injury. These so-called MTDs were found to activate neutrophil migration, and circulating MTDs were suggested to elicit neutrophil-mediated organ injury [23]. Previous studies in liver IR also found that the late phase of IR injury involves migration of neutrophils in response to release of toxic mediators, such as reactive oxygen species and other DAMPs [37]. Based on our results, we hypothesize that MTDs might be one of the cellular DAMPs that play a role in neutrophil activation and in later neutrophil-mediated hepatic IR injury. We found that when the hepatocytes were co-cultured with different concentrations of the MTDs, the percentage of apoptotic cell death decreased, whereas the percentage of necrotic cell death increased in a dose-dependent manner. We also found that the level of TNFα was up-regulated in hepatocytes co-cultured with MTDs (Fig 11). It has been demonstrated stimulation with TNFα can induce either apoptosis or necrosis, and that the late stage of apoptosis is associated with secondary necrotic cell death [38]. These findings suggest that MTDs can lead to both apoptosis and necrosis in hepatocytes, and that late apoptosis might be associated with necrotic cell death in association with TNFα.

Although previous studies have investigated the production of inflammatory cytokines in liver IR models, most of them detected these cytokines at 6h or 24h post-reperfusion, mainly related to the infiltration of white blood cell in the late phases of liver IRI [39–42]. In our study, we found that there was a marked increase of IL-6 and IL-10 just one hour post-reperfusion, during the early phase of liver IRI without neutrophil or monocyte infiltration [37]. The release of inflammatory cytokines in our experiments was likely related to the activation of resident liver macrophages or dendritic cells. Our study further demonstrated that MTDs can trigger apoptosis and necrosis *in vitro* (Fig 4), which may also play a role in the activation of Kupffer cells or dendritic cells to induce an acute inflammatory response. Further studies will determine whether MTDs can contribute to activating immune responses leading to inflammation.

Previous studies have shown MTDs can cause neutrophil-mediated organ injury *in vivo*, associated with significant TNFα and IL-6 secretion [23]. Numerous studies have shown that TLR4 and TLR9 receptors participate *in vivo* in liver IRI models, as well as *in vitro* Kupffer cell models [35, 43–46]. A recent study also demonstrated that MTDs accumulate in the liver, kidneys, lungs, and lymph nodes following tail vein injection, and can induce IL-1β and IL-6 secretion as well as up-regulate numerous proinflammatory genes in the liver [47]. These studies suggest that one of the mechanisms of hepatocyte cell death in our hepatic IR model is through MTDs induction of immune cells. This may include neutrophils, liver resident Kupffer cells, dendritic cells, and infiltrating monocytes, causing inflammation by secreting proinflammatory cytokines. We show clearly in our study that hepatocytes co-cultured with MTDs undergo cell death similar to IR (Fig 3). In this model, we report for the first time that MTDs induce *MyD88* and *NFκB* expression and up-regulate the expression of *TLR2*, *TLR4* and *TLR9* as well as TNFα in the hepatocytes (Figs 10 and 11).

In conclusion, the present study demonstrates convincingly for the first time that MTDs, similar to HMGB1 and nuclear fragment DNA, contribute to hepatic IRI and may serve as a biomarker of liver IRI. Furthermore, our data provides evidence that MTDs play an active role in furthering inflammation and cell death. This study illustrates a novel marker for hepatic IRI and potentially provides additional targets to ameliorate the sterile inflammatory response associated with ischemia and reperfusion injury.
